# Thermal compensation algorithm in ManoScan™ high resolution esophageal manometry: does it really affect manometry metrics and final diagnosis?

**DOI:** 10.55730/1300-0144.6023

**Published:** 2025-06-02

**Authors:** Ekrem ASLAN, Erdem AKBAL

**Affiliations:** 1 Department of Internal Medicine Sciences, Faculty of Medicine, İstinye University, İstanbul, Turkiye; 2 Department of Gastroenterology, Liv Hospital Ulus, İstanbul, Turkiye

**Keywords:** Esophageal manometry, high-resolution manometry, ManoScan™, pressure drift, thermal compensation

## Abstract

**Background/aim:**

The accuracy of pressure measurements with ManoScan™ high-resolution manometry (HRM) catheters decreases due to the development of pressure drift (PD) resulting from variations between room and body temperature. The corrective algorithm called thermal compensation (TC), added to the manometry software program by the manufacturer is used to prevent the development of PD. To date, no studies have demonstrated that PD leads to changes in manometry metrics and/or clinical diagnosis. The present study aims to demonstrate the impact of the TC algorithm on HRM metrics and final diagnosis.

**Materials and methods:**

Records of 124 consecutive esophageal HRM studies with ManoScan™ HRM catheters were retrospectively reviewed. Manometry metrics and final diagnoses were compared by applying formal TC as recommended by the manufacturer (fTC group), without TC (nonTC group), and by performing TC at the 1st s (TC1 group), 5th s (TC5 group), and 10th s (TC10 group), respectively.

**Results:**

Significant differences were observed in values of integrated relaxation pressure (IRP) and distal contractile integral (DCI), and the percentage of weak and intact peristalsis between the fTC group versus the TC1 and TC5 groups. In 28 of 85 studies in which IRP was normal and in 25 of 39 studies in which IRP > 15 mmHg, contrary IRP values were detected when TC was not performed or performed at a different time point than recommended. In the comparison of diagnoses, fewer esophagogastric junction outflow obstruction (EGJ-OO) and more normal esophageal motility (NEM) were diagnosed in the fTC group than in the nonTC group.

**Conclusion:**

The omission of the TC or its application at an incorrect time point on esophageal manometry studies using the ManoScan™ HRM system can lead to inaccurate IRP measurements and diagnostic errors between NEM and EGJ-OO diagnoses.

## 1. Introduction

**Figure 1:**
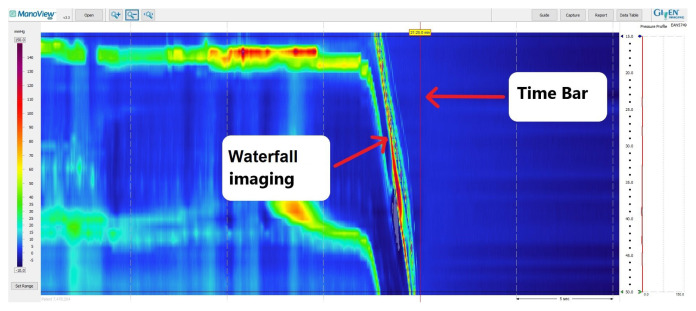
Placement of the vertical time bar on the ManoView™ analysis screen at the end of the “waterfall view” pattern, indicating the point where anatomical pressure ends and atmospheric pressure begins.

The esophageal high-resolution manometry (HRM) procedure is widely used to evaluate esophageal motility disorders and the preoperative assessment of endoscopic or surgical antireflux treatments [1]. For esophageal HRM procedures, the Chicago Classification Working Group’s recently published 4th version (CCv4.0) recommends using solid-state catheters with a sensor spacing of less than two cm [2]. ManoScan™ (Sierra Scientific Instruments, Los Angeles, CA; acquired in 2010 by Given Imaging, Yoqneam, Israeal; purchased in 2014 by Covidien, Dublin, Ireland; and currently Medtronic, Minneapolis, MN) is a widely used esophageal HRM system that uses solid-state catheters consisting of 36 sensors, with each sensor spaced 1 cm apart [3]. However, this system has a significant limitation known as “pressure drift” (PD), which can lead to inaccurate pressure measurements [4]. The manufacturer attributes this issue to the sensors on the esophageal HRM catheter being influenced by body temperature during the procedure [4]. To address this problem, the manufacturer has introduced a corrective algorithm called “thermal compensation” (TC) into the manometry analysis software package (ManoView™). To enable the algorithm’s functionality, TC should be adjusted by pulling the vertical time bar on the ManoView™ analysis screen to the end of the “waterfall image” shape, indicating the point where anatomical pressure ends and atmospheric pressure begins (Figure 1) [5]. In both in vitro and in vivo studies investigating the development of PD in ManoScan™ catheters, it has been shown that factors such as procedure duration, catheter folding, and pressure application to the sensors can influence measurements besides the thermal effect [6–8]. Although TC does not adequately correct factors other than thermal effect, it is used as the only automatic corrective algorithm to prevent PD.

To date, there have been no studies have demonstrated whether PD leads to changes in manometry metrics or clinical diagnosis, or both. We hypothesized that either forgetting to perform TC or conducting it at different time points than recommended could lead to developing PD and impact the final diagnosis. Babei et al. [6] in their experimental study tested PD at 0.5 and 5 s after termination of manometric recording, they found that PD is significantly different at these two time points. Based on this study, we selected the 1st, 5th, and 10th s as alternative time points for TC. This study aims to demonstrate the changes in HRM metrics and final diagnosis when TC is either not performed or is conducted at different time points than recommended by the manufacturer.

## 2. Materials and methods

### 2.1. Study population

A retrospective study was conducted on the records of 136 patients who underwent esophageal HRM using a ManoScan™ solid-state catheter for various indications between January 2018 and May 2023. The study protocol was reviewed and approved by the İstinye University Ethical Committee of Human Research (Meeting number: 2023/07 and Protocol number: 2021/219). The review was conducted using ManoView™ software. The study excluded records of patients in whom the HRM catheter was removed in less than 10 s after removal from the body, records with a study duration exceeding 15 min, studies in which the catheter was folded during the procedure, and records of patients in whom pressure was applied to the sensors on the catheter during catheter insertion or removal.

### 2.2. Equipment

For all patients who underwent manometry, ManoScan™ solid-state catheters (model A120) with 36 pressure sensors placed at 1 cm intervals and with a diameter of 2.75mm or above were utilized. Each catheter had been used less than 200 times and was within the 2-year warranty period. Prior to each procedure, the catheter was calibrated for pressure and temperature in accordance with the manufacturer’s recommendations. The ManoShield™ disposable catheter sheath was used in all procedures.

### 2.3. HRM procedure

The HRM procedure was conducted in all patients following a 6-h fasting period. Following the administration of topical lidocaine, the ManoScan™ solid-state catheter was transnasally placed. Patients were then positioned supine and administered 10 sequential 5 mL water swallows. Subsequently, a 250 mL rapid swallow test was performed while patients were in an upright position. After the swallows had been completed, the operator removed the HRM catheter, suspended it in the air at the bed level, and concluded the study using the software.

### 2.4. Data collection

**Figure 2:**
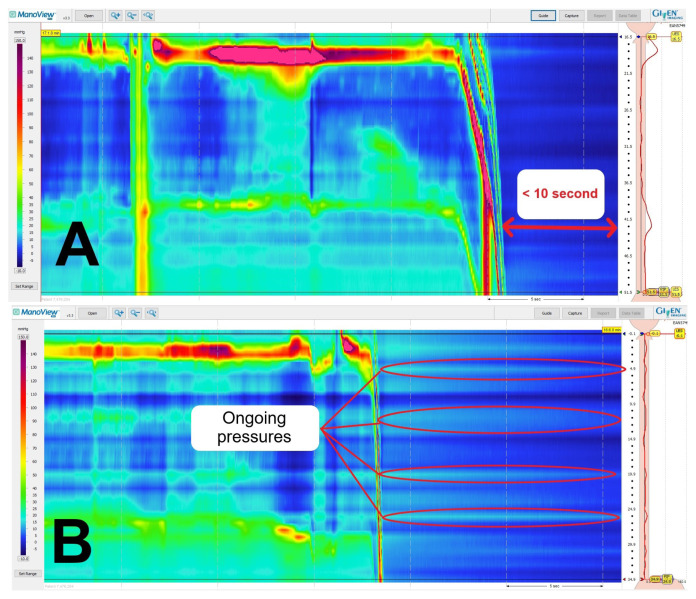
Examples show recording time less than 10 s after the ManoScan™ HRM catheter was removed from the body (A) and pressure remaining on the sensors after the study was completed (B).

Data recorded for each HRM study from 136 patients were individually reviewed using the ManoView™ software. A total of 12 recordings, which had a record duration of less than 10 s after catheter removal or showed ongoing pressure on the sensors, were excluded from the study (Figures 2A and 2B). Patient age, sex, and indications for HRM were recorded. All HRM studies were evaluated by the same reviewer using the ManoView™ software. For each HRM study, pressure corrections were applied in the following order using the vertical time bar. First, corrections were made without performing TC. After that, TC was performed at the point where the waterfall image concluded, following the manufacturer’s recommendations. Finally, the study returned to the beginning each time to apply TC again at time points of 1 s, 5 s, and 10 s after the end of the waterfall image. Topographic pressure measurement values [upper esophageal sphincter pressure (UESP), lower esophageal sphincter pressure (LESP), integrated relaxation pressure (IRP), distal latency (DL), and distal contractile integral (DCI)] obtained at the end of all studies, contraction strength (failed, weak, and ineffective), contraction pattern (premature, fragmented, and intact), intrabolus pressure pattern (panesophageal pressurization), and the final diagnosis resulting from the analysis were recorded separately for each study.

### 2.5. Definitions

According to the TC timing, HRM metrics in the examined manometry records and clinical diagnoses were categorized as follows: 1-Those without TC: nonTC group; 2-Those who underwent TC at the recommended time by the manufacturer: formal TC (fTC) group; 3-Those who underwent TC at 1st second: TC1 group; 4-Those who underwent TC at 5th second: TC5 group; and 5-Those who underwent TC at 10th second: TC10 group. After evaluation of HRM studies: 1-Elevated median IRP (>15 mmHg) and 100% failed peristalsis were defined as type 1 achalasia (T1A); 2-Elevated median IRP (>15 mmHg), 100% failed peristalsis, and panesophageal pressurization with ≥20% of swallows were defined as type 2 achalasia (T2A); 3-Elevated median IRP (> 15 mmHg), no normal peristalsis, and premature (spastic) contractions with DCI > 450 mmHg·s·cm in ≥20% of swallows were defined as type 3 achalasia (T3A); 4-Elevated median IRP (>15 mmHg) and sufficient evidence of peristalsis such that criteria for types I–III achalasia are not met were defined as esophagogastric outflow obstruction (EGJ-OO); 5-Normal median IRP and 100% failed peristalsis were defined as absent contractility (AC); 6-Normal median IRP and ≥20% premature contractions with DCI > 450 mmHg·s·cm were defined as distal esophageal spasm (DES); 7-At least two swallows with DCI > 8000 mmHg·s·cm was defined as hypercontractile esophagus (HE); 8-More than 50% of ineffective swallows were defined as ineffective esophageal motility (IEM) 9-More than 50% of fragmented contractions with DCI > 450 mmHg·s·cm were defined as fragmented peristalsis (FP); 10-If IRP values are borderline and there is evidence of esophageal pressurization with 100% failed peristalsis were defined as achalasia cannot rule out (ACR). HRM studies that did not meet the above classifications were defined as normal esophageal motility (NEM) [9].

### 2.6. Statistical analysis

The statistical analysis was carried out using SPSS 23.0 (SPSS Inc., Chicago, IL). Data were expressed as mean ± standard deviation for normally distributed data, while the median (interquartile range) was used for nonnormally distributed data. The normality tests were conducted with Kolmogorov-Smirnov tests; the distribution was assumed normal when both tests produced p values >0.05.

Statistical comparisons of individual groups were based on independent t-tests for continuous and normally distributed variables. Mann-Whitney U and Kruskal-Wallis tests were implemented when the distribution was not normal. If Kruskal-Wallis revealed any differences, the source of differences was investigated with Mann-Whitney U, and the means having differences were allocated the letters *k, l*, and *m*. The relationship between categorical variables was investigated using the Chi-square.

## 3. Results

Of the 124 patients enrolled in the study and whose data were analyzed, the mean age was 45.15 ± 15.08 years, with 54 (43.5%) male and 70 (56.5%) female. Manometric examination was conducted based on indications such as dysphagia in 105 (84.7%) patients, chest pain in 3 (2.4%), gastroesophageal reflux disease in 3 (2.4%), preoperative evaluation for antireflux surgery in 9 (7.3%), and preoperative assessment for endoscopic reflux treatment in 4 (3.2%) (Table 1).

In the data related to topographical pressure measurements such as UESP, LESP, and DL, and in the percentages reflecting contraction force and patterns such as failed, ineffective, pressurization, premature, and fragmented contractions, no significant differences were found among the fTC, nonTC, TC1, TC5, and TC10 groups (p > 0.05). However, significant differences were observed among the groups based on TC timing in topographical pressure measurements. These changes were evident in the IRP (p = 0.029) and DCI (p = 0.002) values, as well as percentages of weak (p = 0.039) and intact (p = 0.049) peristalsis patterns (Table 2). The topographical pressure measurements showed that the fTC-IRP value was 11.25 mmHg, which was significantly higher than the TC1-IRP value of 8.45 mmHg and the TC5-IRP value of 9.70 mmHg (p = 0.008, p = 0.047, respectively). In the TC10 group, the DCI value was 1682.70 mmHg·s·cm, which was significantly higher than the fTC-DCI value of 1107.40 mmHg·s·cm the nonTC-DCI value of 1064.10 mmHg·s·cm and the TC1-DCI value of 1134.15 mmHg·s·cm (p = 0.002, p = 0.001, p = 0.001, respectively).

The percentage of weak contractions, derived from data indicating contraction force, was statistically significantly lower in the TC10 group compared to the nonTC, TC1, and fTC groups (p = 0.004, p = 0.023, and p = 0.038, respectively). Furthermore, the percentage of intact contractions, derived from data indicating contraction patterns, was higher in the TC10 group than in the nonTC and TC1 groups, with the difference being statistically significant (p = 0.005 and p = 0.040, respectively). Similarly, in the TC5 group, the percentage of intact contractions was higher compared to the nonTC group, and the difference was statistically significant (p = 0.047).

When evaluating HRM metrics in subgroups with higher patient distribution, such as NEM, EGJ-OO, IEM, and achalasia (Types 1, 2, and 3), it was observed that only in the EGJ-OO subgroup, the DCI value in the TC10 group was statistically higher compared to the fTC, nonTC, and TC1 groups [TC10: 3051 mmHg vs. fTC: 1665.10 mmHg vs. nonTC: 1573 mmHg vs. TC1: 1857.65 mmHg, p = 0.002, p = 0.001, p = 0.048)]. No significant differences over time were observed among other metrics in the subgroups (Tables 3, 4, 5, and 6).

**Figure 3:**
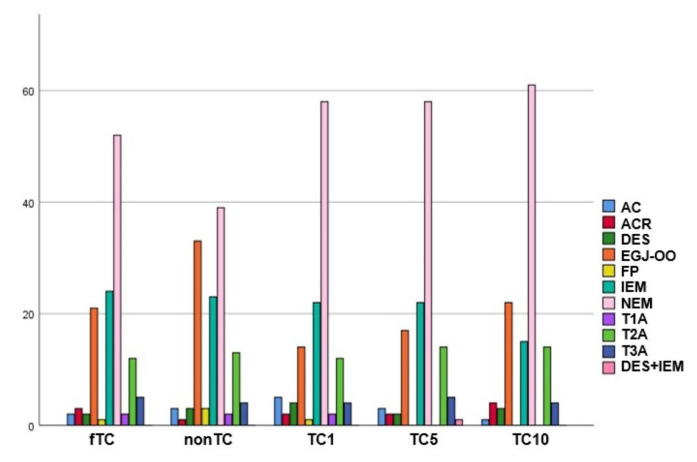
There is a significant difference between the nonTC and TC1 groups in patients diagnosed with EGJ-OO (n = 33 vs. 14, p = 0.019) and between the nonTC and TC10 groups in patients diagnosed with NEM (n = 39 vs. 61, p = 0.038). AC: absent contractility, ACR: achalasia cannot roule out, DES: distal esophageal spasm, EGJ-OO: esophagogastric junction outflow obstruction, FP: fragmented peristaltis, IEM: ineffective esophageal motility, NEM: normal esophageal motility, T1A: Type 1 achalasia, T2A: Type 2 achalasia, T3A: Type 3 achalasia

Upon analyzing the study groups for diagnostic changes related to AC, ACR, DES, FP, IEM, and achalasia (Types 1, 2, and 3), it was observed that there were no significant changes based on the performance and timing of TC (Table 7). Although the fTC group did not create a statistically significant difference compared to the nonTC group in terms of diagnostic changes, it was noted that there were fewer patients diagnosed with EGJ-OO (21 vs. 33, p > 0.05) and more patients diagnosed with NEM in the fTC group (52 vs. 39, p > 0.05). Among the groups, the number of patients diagnosed with EGJ-OO and NEM differed significantly between the nonTC and TC1 groups and between the nonTC and TC10 groups (Figure 3). Accordingly, it was found that in the nonTC group, significantly more diagnoses of EGJ-OO were made compared to the TC1 group (n = 33 vs. 14, p = 0.019). Additionally, no significant difference in the number of patients diagnosed with EGJ - OO was observed among the TC groups. Furthermore, in the TC10 group, there was a significantly higher number of patients diagnosed with NEM compared to the nonTC group (n = 39 vs. 61, p = 0.038). However, no significant difference in the number of patients diagnosed with NEM was observed among the TC groups.

In the fTC group, IRP was initially measured as < 15 mmHg in 85 studies; in 26 of these, IRP increased to >15 mmHg when the timing of TC was changed. Conversely, in 39 studies within the same group where IRP was initially measured as >15 mmHg, changing the TC timing resulted in IRP dropping below 15 mmHg in 25 study. The changes in IRP and diagnosis according to TC timing are detailed in Table 8.

## 4. Discussion

This study is the first to investigate the impact of PD and TC timing on manometry metrics and final diagnoses in patients undergoing manometry using the ManoScan™ esophageal HRM system.

The warming of pressure sensors on ManoScan™ HRM catheters often results in PD secondary to the thermal expansion of circumferential metallic sensors after catheter removal [10]. The electromechanical mechanism leading to PD in HRM sensors is not fully understood, but it is thought to be related to the sensor’s structure, which consists of a rigid inner electrode with an air gap in between and an outer sensing membrane that is prone to deformation [11].

TC is a corrective algorithm integrated into the post-procedural ManoView™ software by the manufacturer to prevent PD development due to thermal effects in manometry studies conducted with ManoScan™ esophageal catheters [5]. Previous studies have shown that thermal effects, the duration of the procedure, catheter folding, and pressure applied to the catheter can lead to PD, and performing TC alone may not be sufficient to prevent PD development [7,8,11]. However, to date, all studies have focused on the development of PD, and there is no available literature data on the clinical effects of observed PD in the interpretation of manometry studies, regardless of whether corrective algorithms are applied or not.

Our study results indicate PD development in the upper esophageal sphincter (UES) and lower esophageal sphincter (LES) resting pressures, represented by UESP and LESP values, when TC correction is not performed or TC is performed at a different time point than recommended (UESP: between −1.35 and +6.20 mmHg, LESP: between −2 and −0.2 mmHg). However, the difference in pressure was not statistically significant. According to Babaei et al. [7], exposure to average pressure in high-pressure zones of UES and LES is the most crucial determinant of PD development, which could impact the clinical interpretation of HRM studies. Nevertheless, according to our study results, PD development does not lead to a statistically significant difference in the clinical interpretation of UES and LES pressures.

The IRP is defined as the average of the for second maximum relaxation of LES within a ten second swallowing window, starting from UES relaxation with reference to intragastric pressure [9]. According to our study results, when TC correction was performed at 1 s and 5 s, the median IRP value was significantly lower compared to TC performed at the recommended time. While the differences in IRP values between the fTC group versus the TC1 and TC5 groups are statistically significant, it does not lead to a significant diagnostic inaccuracy. Fass et al. [12] showed that IRP values could vary significantly based on the sensor chosen as a reference within the stomach, leading to inconsistent diagnoses in 28% of HRM studies. In our study, we believe that changes in gastric sensor pressure due to the TC condition cause these erroneous measurements in IRP values.

The IRP is a fundamental HRM metric used to assess the adequacy of relaxation in the esophagogastric junction (EGJ) during swallowing, with a median threshold value of 15 mmHg determined for ManoScan™ esophageal HRM systems in the supine position [13,14]. The median IRP value serves as the diagnostic algorithm’s main criterion and the initial point of differentiating EGJ outflow disorders from disorders of peristalsis [2]. In our study, we observed that in 30% of HRM studies with a normal IRP when fTC was performed, the IRP exceeded 15 mmHg when the TC timing was altered. Furthermore, in 29% of these cases, this change in IRP led to a shift in diagnosis from NEM to EGJ-OO.

EGJ-OO is a heterogeneous group of disorders (including mechanical, functional, medication-related, and artifactual causes) defined as impaired relaxation of the esophagogastric junction and elevated IRP more than 15 mmHg on HRM, but with preserved esophageal peristalsis. In patients diagnosed with EGJ-OO, manometry alone is insufficient to establish the diagnosis, and further diagnostic work-up, including endoscopy, barium esophagography, endoscopic ultrasonography, thoracic CT, and functional luminal imaging probe studies, is required [15]. Therefore, misdiagnosis of patients as having EGJ-OO instead of NEM due to errors in TC timing could lead to wasting of time and resources.

In CCv4.0, other crucial HRM metric that play a key role in diagnosing esophageal HRM studies is DCI, indicating the strength of esophageal body contraction [14]. Our study results suggest a tendency for DCI values to be lower when TC is not performed. However, when TC is performed at a different time point than recommended, there is an increasing trend in DCI values as the duration of TC increases. The most significant increase was observed when TC correction was performed at the 10th s, leading to a statistically significant difference compared to TC at the recommended time. Subgroup analysis revealed that studies diagnosed with EGJ-OO were the primary diagnostic group contributing to this difference. However, these significant changes in the DCI values depending on the TC timing do not lead to a change in final diagnosis.

Another important result shown by our study is that there was no statistically significant difference in terms of diagnostic change when TC correction was not made or when TC was performed at a different time point than recommended. Parthasarathy et al. [5], in their study using the ManoScan™ anorectal HRM (HRAM) catheter, showed that the TC algorithm alone partially prevented PD development, but developing PD rarely caused a change in diagnosis (diagnostic change in 8 patients out of 76), consistent with our study results.

Data on the propensity of developing PD with systems using solid-state catheters other than the ManoScan™ HRM system are limited. A study by Elangovan et al. [16] involving patients diagnosed with NEM and EGJ-OO, using solid-state HRM catheter (Laborie Medical Technologies, Quebec City, Canada) for measurements, showed a significant pressure decrease ranging from 19% to 40% between the first and tenth swallows in both groups. Although the authors hypothesized that the decrease in IRP might be due to a longer adaptation period of the LES to the catheter or catheter repositioning or migration with each swallow, we believe it could also be related to the development of PD over time.

We acknowledge that our study has several limitations. First, the study was designed retrospectively, and most of the cases that underwent HRM were presenting with complaints of dysphagia. We believe that a comparative analysis with a healthy control group would provide a more accurate reflection of the impact of TC on PD. The second limitation is that all HRM tracings were interpreted by a single physician, without interobserver validation, potentially introducing observer bias in the assessment. Thirdly, the lack of comparative analysis across different HRM catheter systems, such as the Solar GI HRM system (Medical Measurement Systems, Enschede, The Netherlands) and The Starlet™ HRM system (Star Medical Inc., Tokyo, Japan), both of which do not require TC, restricts the external validity of our conclusions and underscores the need for future comparative studies involving various catheter technologies.

In conclusion, our study represents the first clinical investigation examining the effects of PD on HRM metrics and final HRM diagnoses. The omission of the TC algorithm or its application at a different time point can lead to the development of PD, which affects the IRP values and may result in the incorrect diagnosis of EGJ-OO in individuals with normal esophageal motor function. Comparative studies with other HRM systems that do not require TC correction are needed to investigate the extent of the effect of TC correction on clinical diagnosis and HRM metrics.

## Figures and Tables

**Table 1:** Patient demographics.

**Age (year) (mean ± SD)**	45.15 ± 15.08
**Sex (Male/Female) (n,%)**	54 (43.5%)/70 (56.5%)
**Indications (n,%)**	
Dysphagia	105 (84.7%)
Chest pain	3 (2.4%)
Gastroesophageal reflux disease	3 (2.4%)
Preoperative evaluation for ARS	9 (7.3%)
Preoperative assessment for ERT	4 (3.2%)

ARS: Antireflux surgery, ERT: Endoscopic reflux treatment

**Table 2:** HRM metrics according to TC timing.

	fTC	nonTC	TC1	TC5	TC10	p value
**UESP (mmHg)**	81.5 (54.1–111.2)*	80.2 (54.5–112.3)*	82.4 (54.4–111.4)*	83.5 (58.9–115)*	87.75 (61.8–117.6)*	0.567 a
**LESP (mmHg)**	24.3 (17.6–37.5)*	24.1 (17.6–38.2)*	22.3 (15.1–33.7)*	22.6 (15.3–33.3)*	24 (14.6–34.9)*	0.350 a
**IRP (mmHg)**	11.2 (7.7–20.4) *.k.l	12.1 (5.9–20.6) *^m^	8.4 (5.2–14.9) * k. m	9.7 (6.3–16.2) * l	10.5 (6.4–16.8)*	**p = 0.029** a **pk = 0.008** **pl = 0.047** **pm = 0.012**
**Swallowing**						
Weak (%)	0 (0–10)*.^k^	0 (0–20)*.^l^	0 (0–10)*.^m^	0 (0–10)*	0 (0–0)*. k.l.m	**p = 0.039** a **pk = 0.038** **pl = 0.004** **pm = 0.023**
Failed (%)	0 (0–60)*	10 (0–70)*	0 (0–60)*	0 (0–57.50)*	0 (0–50)*	0.841 a
Ineffective (%)	20 (0–90)*	30 (0–100)*	20 (0–90)*	10 (0–87.50)*	10 (0–80)*	0.122 a
Pressurisation (%)	0 (0–10)*	0 (0–0)*	0 (0–10)*	0 (0–10)*	0 (0–20)*	0.426 a
Premature (%)	0 (0–0)*	0 (0–0)*	0 (0–0)*	0 (0–0)*	0 (0–0)*	0.596 a
Fragmented (%)	0 (0–0)*	0 (0–0)*	0 (0–0)*	0 (0–0)*	0 (0–0)*	0.088 a
Intact (%)	75 (10–100)*	55 (0–100)*.k.l	70 (0–100)*.^m^	80 (10–100)*.^k^	90 (12.5–100)*.l.m	**p = 0.049** a **pk = 0.047** **pl = 0.005** **pm = 0.040**
**DL (s)**	6.2 (5.4–7)*	6.2 (5.4–6.9)*	6.2 (5.4–7)*	6.1 (5.4–6.8)*	5.9 (5.3–6.7)*	0.551 a
**DCI (mmHg**·**s**·**cm)**	1107.4 (685.7–2004.5)*.^k^	1064.1 (480.9–1990.5)*.^l^	1134.1 (642.8–1962.1)*.^m^	1324 (793.8–2294.3)*	1682.7 (987.2–2616.9)*.k.l.m	**p = 0.002** a **pk = 0.002** **pl = 0.001** **pm = 0.001**

* Median (IQR);

a Kruskal-Wallis Test;

k, l, m Letters indicating statistical difference based on Mann-Whitney U test.

HRM: High resolution manometry, TC: Thermal compensation, DCI: Distal contractile integral, DL: Distal latency, IRP: Integrated relaxation pressure, LESP: Lower esophageal sphincter pressure, UESP: Upper esophageal sphincter pressure.

**Table 3:** HRM metrics in EGJ - OO patients according to TC timing

	fTC	nonTC	TC1	TC5	TC10	p value
**UESP (mmHg)**	72.7 (45.5–105.4)^*^	78.5 (54.1–112.7)^*^	71.7 (52–106.7)^*^	76.7 (50.3–117.6)^*^	68.5 (51.5–102.9)^*^	0.974 a
**LESP (mmHg)**	43.4 (33.5–50.8)^*^	41.1 (26.9–53.3)^*^	40.5 (27.6–49.7)^*^	42 (29.4–45.5)^*^	37.8 (28.5–52.8)^*^	0.848 a
**IRP (mmHg)**	21.60 (18.2–26.6)^*^	19.10 (17–24.6)^*^	17.55 (15.9–25.4)^*^	16.70 (16.1–23.6)^*^.^l^	18.15 (15.9–22)^*^	0.105 a
**Swallowing**						
Weak (%)	0 (0–10)^*^	0 (0–20)^*^	0 (0–5)^*^	0 (0–0)^*^	0 (0–0)^*^	0.243 a
Failed (%)	0 (0–40)^*^	0 (0–40)^*^	10 (0–52.5)^*^	0 (0–40)^*^	0 (0–12.5)^*^	0.699 a
Ineffective (%)	0 (0–60)^*^	10 (0–80)^*^	10 (0–70)^*^	0 (0–65)^*^	0 (0–25)^*^	0.647 a
Pressurisation (%)	0 (0–10)^*^	0 (0–0)^*^	0 (0–22.5)^*^	0 (0–25)^*^	0 (0–12.5)^*^	0.420 a
Premature (%)	0 (0–10)^*^	0 (0–0)^*^	0 (0–10)^*^	0 (0–15)^*^	0 (0–12.5)^*^	0.432 a
Fragmented (%)	0 (0–0)^*^	0 (0–0)^*^	0 (0–0)^*^	0 (0–0)^*^	0 (0–0)^*^	0.888 a
Intact (%)	90 (40–100)^*^	80 (15–100)^*^	40 (10–100)^*^	80 (30–100)^*^	95 (57.5–100)^*^	0.355 a
**DL (s)**	5.80 (5.2–6.8)^*^	6 (5.2–7.0)^*^	5.80 (5.07–7.1)^*^	6.10 (4.9–6.9)^*^	5.90 (4.9–6.9)^*^	0.979 a
**DCI (mmHg**·**s**·**cm)**	1665.1 (998.8–2348.2)^*^.^k^	1573 (649.1–2813.4)^*^.^l^	1857.6 (955.1–4374.3)^*^.^m^	2429.3 (1394.8–152.6)^*^	3051 (2371.9–5848.3)^*^.k.l.m	**p = 0.005** a **pk = 0.002** **pl = 0.001** **pm = 0.048**

Median (IQR);

a Kruskal-Wallis Test;

k, l, m Letters indicating statistical difference based on Mann-Whitney U test.

HRM: High resolution manometry, TC: Thermal compensation, DCI: Distal contractile integral, DL: Distal latency, IRP: Integrated relaxation pressure, LESP: Lower esophageal sphincter pressure, UESP: Upper esophageal sphincter pressure.

**Table 4:** HRM metrics in IEM patients according to TC timing.

	fTC	nonTC	TC1	TC5	TC10	p value
**UESP (mmHg)**	91.3 (61.2–110.8)*	94 (65.7–110.8)*	92.6 (62.2–111.2)*	85.4 (67.5–112.1)*	95.7 (65.6–106.5)*	0.991 a
**LESP (mmHg)**	19.1 (13.6–24)*	14.7 (9.7–22.1)*	15 (11.1–23.4)*	16.4 (11.4–22)*	17.6 (13.5–25.8)*	0.695 a
**IRP (mmHg)**	8.5 (5.7–11.1)*	8 (3.8–10.1)*	7.2 (4.4–8.6)*	7 (4.4–10.7)*	7.2 (5.4–11.7)*	0.4865 a
**Swallowing**						
Weak (%)	35 (10–67.5)*	40 (10–60)*	45 (20–62.50)*	30 (10–45)*	20 (0–60)*	0.569 a
Failed (%)	45 (20–77.5)*	40 (20–80)*	45 (20–70)*	30 (17.50–70)*	60 (30–90)*	0.500 a
Ineffective (%)	90 (80–100)*	100 (90–100)*	90 (80–100)*	80 (60–92.50)*	90 (80–100)*	0.128 a
Pressurisation (%)	0 (0–7.5)*	0 (0–0)*	0 (0–0)*	0 (0–2.50)*	0 (0–70)*	0.701 a
Premature (%)	0 (0–0)*	0 (0–0)*	0 (0–0)*	0 (0–0)*	0 (0–0)*	0.532 a
Fragmented (%)	0 (0–0)*	0 (0–0)*	0 (0–0)*	0 (0–0)*	0 (0–0)*	0.256 a
Intact (%)	10 (0–17.5)*	0 (0–10)*	10 (0–20)*	15 (7.50–40)*	10 (0–20)*.	0.095 a
**DL (s)**	6.6 (5.7–7.7)*	6.80 (5.50–7.20)*	6.50 (5.85–7.32)*	6.10 (5.40–6.82)*	6 (4.90–6.70)*	0.212 a
**DCI (mmHg**·**s**·**cm)**	349 (227.3–457.9)*	288.70 (202.10–438.10)*	329.95 (218.30–454.50)*	425.20 (331.12–541.50)*	403.50 (292.50–723.10)*	0.162 a

* Median (IQR);

a Kruskal-Wallis Test;

k, l, m Letters indicating statistical difference based on Mann-Whitney U test.

HRM: High resolution manometry, TC: Thermal compensation, DCI: Distal contractile integral, DL: Distal latency, IRP: Integrated relaxation pressure, LESP: Lower esophageal sphincter pressure, UESP: Upper esophageal sphincter pressure.

**Table 5:** HRM metrics in NEM patients according to TC timing.

	fTC	nonTC	TC1	TC5	TC10	p value
**UESP (mmHg)**	89 (59–112.9)*	83.2 (54.6–112.6)*	89.9 (56.8–112.5)*	90.9 (61.2–119.4)*	98.6 (70.4–123.3)*	0.496 a
**LESP (mmHg)**	20.9 (16.2–25.9)*	19.6 (14–22.2)*	19.6 (15–25.6)*	20.8 (15.1–26.2)*	19.1 (12.7–25.9)*	0.512 a
**IRP (mmHg)**	8.9 (6.4–11.9)*	7.1 (4.3–9.9)*	7.3 (3.7–10)*	7.4 (3.9–10.2)*	7.3 (3.6–10.3)*	0.232 a
**Swallowing**						
Weak (%)	0 (0–0)*	0 (0–10)*	0 (0–10)*	0 (0–0)*	0 (0–0)*	0.689 a
Failed (%)	0 (0–0)*	0 (0–0)*	0 (0–0)*	0 (0–0)*	0 (0–0)*	0.904 a
Ineffective (%)	0 (0–10)*	0 (0–10)*	0 (0–10)*	0 (0–10)*	0 (0–10)*	0.796 a
Pressurisation (%)	0 (0–0)*	0 (0–0)*	0 (0–0)*	0 (0–0)*	0 (0–0)*	0.702 a
Premature (%)	0 (0–0)*	0 (0–0)*	0 (0–0)*	0 (0–0)*	0 (0–0)*	0.255 a
Fragmented (%)	0 (0–0)*	0 (0–0)*	0 (0–0)*	0 (0–0)*	0 (0–0)*	0.094 a
Intact (%)	100 (82.5–100)*	100 (80–100)*	100 (80–100)*	100 (87.5–100)*	100 (90–100)*	0.355 a
**DL (s)**	6.3 (5.7–7)*	6.3 (5.6–6.8)*	6.30 (5.7–7.1)*	6.1 (5.6–7)*	6.1 (5.5–6.9)*	0.920 a
**DCI (mmHg**·**s**·**cm)**	1278.1 (971.3–2241)*	1402.9 (848.5–2228.8)*	1306.8 (905.6–2075.8)*	1542.6 (1014.6–2231.9)*	1478.9 (1031.8–2277.5)*	0.565 a

* Median (IQR);

a Kruskal-Wallis Test;

k, l, m Letters indicating statistical difference based on Mann-Whitney U test.

HRM: High resolution manometry, TC: Thermal compensation, DCI: Distal contractile integral, DL: Distal latency, IRP: Integrated relaxation pressure, LESP: Lower esophageal sphincter pressure, UESP: Upper esophageal sphincter pressure.

**Table 6:** HRM metrics in Achalasia patients according to TC timing.

	fTC	nonTC	TC1	TC5	TC10	p value
**UESP (mmHg)**	75.6 (57.1–130.4)*	76.7 (54.3–127.7)*	81.4 (60.1–124.5)*	76.9 (60.6–125.6)*	88.5 (63.6–131.3)*	0.945 a
**LESP (mmHg)**	40.3 (32.7–49.8)*	37.7 (30–44.8)*	37.1 (28.7–44.2)*	37.6 (29.6–47.9)*	36.7 (29.7–43.7)*	0.819 a
**IRP (mmHg)**	26.2 (23.2–34.9)*	26.2 (23.3–29.4)*	24.6 (21.3–35.1)*	24.6 (22.4–29.4)*	24.7 (22.3–30.5)*	0.950 a
**Swallowing**						
Weak (%)	0 (0–0)*	0 (0–0)*	0 (0–0)*	0 (0–0)*	0 (0–0)*	1.000 a
Failed (%)	100 (40–100)*	100 (100–100)*	100 (70–100)*	100 (80–100)*	100 (77.50–100)*	0.939 a
Ineffective (%)	100 (40–100)*	100 (100–100)*	100 (70–100)*	100 (80–100)*	100 (70–100)*	0.936 a
Pressurisation (%)	100 (30–100)*	70 (40–100)*	85 (47.50–100)*	100 (40–100)*	100 (70–100)*	0.237 a
Premature (%)	0 (0–20)*	0 (0–0)*	0 (0–2.5)*	0 (0–20)*	0 (0–5)*	0.956 a
Fragmented (%)	0 (0–0)*	0 (0–0)*	0 (0–0)*	0 (0–0)*	0 (0–0)*	0.096 a
Intact (%)	0 (0–60)*	0 (0–0)*	0 (0–30)*	0 (0–20)*	0 (0–5)*	0.863 a
**DL (s)**	3.8 (0.5–4.2)*	3.4 (2.5–4.1)*	3.9 (2.5–5.4)*	3.8 (2.6–4.1)*	3.55 (2.5–4)*	0.951 a
**DCI (mmHg**·**s**·**cm)**	1670.8 (1094.5–3443.2)*	1382.5 (957.1–2330.2)*	1755.4 (992.4–2225.8)*	1928.2 (1111.6–4628)*	2030.8 (1189–2951)*	0.807 a

* Median (IQR);

a Kruskal - Wallis Test;

k, l, m Letters indicating statistical difference based on Mann - Whitney U test.

HRM: High resolution manometry, TC: Thermal compensation, DCI: Distal contractile integral, DL: Distal latency, IRP: Integrated relaxation pressure, LESP: Lower esophageal sphincter pressure, UESP: Upper esophageal sphincter pressure.

**Table 7:** Distribution of final diagnosis according to timing of TC.

Diagnosis	fTC	nonTC	TC1	TC5	TC10	p value*
**AC (n, %)**	2 (1.6%)	3 (2.4%)	5 (4%)	3 (2.4%)	1 (0.8%)	0.510
**ACR (n, %)**	3 (2.4%)	1 (0.8%)	2 (1.6%)	2 (1.6%)	4 (3.2%)	0.688
**DES (n, %)**	2 (1.6%)	3 (2.4%)	4 (3.2%)	2 (1.6%)	3 (2.4%)	0.908
**EGJ-OO (n, %)**	21 (16.9%)	33 (26.6%) k	14 (11.3%) k	17 (13.7%)	22 (17.7%)	0.019
**FP (n, %)**	1 (0.8%)	3 (2.4%)	1 (0.8%)	0 (0%)	0 (0%)	0.156
**IEM (n, %)**	24 (19.4%)	23 (18.5%)	22 (17.7%)	22 (17.7%)	15 (12.1%)	0.576
**NEM (n, %)**	52 (41.9%)	39 (31.5%)^l^	58 (46.8%)	58 (46.8%)	61 (49.2%)^l^	0.038
**T1A (n, %)**	2 (1.6%)	2 (1.6%)	2 (1.6%)	0 (0%)	0 (0%)	0.187
**T2A (n, %)**	12 (9.7%)	13 (10.5%)	12 (9.7%)	14 (11.3%)	14 (11.3%)	0.987
**T3A (n, %)**	5 (4%)	4 (3.2%)	4 (3.2%)	5 (4%)	4 (3.2%)	0.991
**DES+IEM (n, %)**	0 (0%)	0 (0%)	0 (0%)	1 (0.8%)	0 (0%)	0.597

* Chi-Square Test;

k, l Letters indicating statistical difference based on Adjusted Residuals.

AC: Absent contractility, ACR: Achalasia cannot roule out, DES: Distal esophageal spasm, EGJ-OO: Esophagogastric junction outflow obstruction, FP: Fragmented peristaltis, IEM: Ineffective esophageal motility, NEM: Normal esophageal motility, T1A: Type 1 achalasia, T2A: Type 2 achalasia, T3A: Type 3 achalasia.

**Table 8:** Change in IRP and final diagnosis according to TC timing.

	fTC group IRP < 15mmHg (n:85)					fTC group IRP > 15mmHg (n: 39)				
	NEM	IEM	EGJ-OO	Achlasia	Other	NEM	IEM	EGJ-OO	Achalasia	Other
**fTC**	53	24	0	0	8	0	0	21	18	0
**nonTC**	38	22	14	2	9	1	1	20	17	0
**TC1**	52	22	0	0	11	7	0	14	17	1
**TC5**	52	21	3	1	8	6	1	14	18	0
**TC10**	55	14	7	1	8	6	1	15	17	0

EGJ-OO: Esophagogastric junction outflow obstruction, IEM: Ineffective esophageal motility, NEM: Normal esophageal motility, TC: Thermal compensation, Other (absent contractility, achalasia cannot roule out, distal esophageal spasm, fragmented peristaltis).
